# Empowering Young Adults as Agents of Household Dietary Change: Findings From a Pilot Study Involving a Digital, Family-Led Sodium Reduction Intervention in Singapore

**DOI:** 10.1016/j.cdnut.2025.107523

**Published:** 2025-08-05

**Authors:** Kimberly Mei Yi Low, Cindy Mei Jun Chan, Ian Yi Han Ang, Mary Foong-Fong Chong, Shahmir H Ali

**Affiliations:** Saw Swee Hock School of Public Health, National University of Singapore, Singapore, Singapore

**Keywords:** young adult, family, sodium, dietary, health behavior, multigenerational, Asia, restaurants, theory of planned behavior, digital health

## Abstract

**Background:**

Unhealthy dietary habits such as high-sodium intake, are socially embedded and often resistant to individual-level interventions. Family-led approaches, where 1 member initiates change within the household, may offer a more effective alternative.

**Objectives:**

This pilot study assessed the feasibility and preliminary impact of a digitally delivered, young adult-led sodium reduction intervention on household-level knowledge, attitudes, and behaviors in Singapore.

**Methods:**

In a pre-post, single-group design, young adults completed a co-created, self-paced online course featuring video lessons, interactive assignments, and personalized feedback. Over 2 weeks, participants developed sodium-reduction goals and implemented them through tailored 4-wk action plans. Weekly reflections and course metadata captured goal progress, effort, strategies, and barriers. Family members completed parallel preintervention and postintervention surveys, although they did not receive the intervention directly. Surveys assessed constructs from the Theory of Planned Behavior. Multivariable linear mixed models evaluated changes over time, adjusting for demographic and health characteristics.

**Results:**

Overall, 35 young adults (mean age: 24.4 y; standard deviation [SD]: 3.1) and 79 family members (mean age: 43.0 y; SD: 15.5) completed the intervention. Young adults took a mean of 7.7 d to complete the course, with most crafted goals focusing on reducing sodium intake when eating out. Participants reported higher effort and success with personal goals than family-oriented ones. Perceived behavioral control showed the greatest improvement among both young adults (+2.64, 95% confidence interval [95% CI]: 2.05, 3.22) and family members (+1.82, 95% CI: 1.42, 2.22). Significant gains were also observed in knowledge, behaviors, subjective norms, and behavioral intentions for all participants (all *P* < 0.001). Engagement metrics (e.g., time spent on the course, effort put into the action plans) were not associated with differential changes in most outcomes.

**Conclusions:**

A young adult-led, family-focused digital intervention was feasible and demonstrated preliminary improvements in household sodium-related outcomes, warranting further evaluation in larger, more diverse populations.

## Introduction

Excessive sodium intake has emerged as a critical public health issue due to its strong correlations with noncommunicable diseases (NCDs), including hypertension and cardiovascular diseases, which are major contributors to global disease burdens and consistently the leading causes of mortality worldwide [1]. Approximately 1.9 million deaths are attributed to excessive sodium intake yearly [2] with the prevalence of hypertension surging by 144% in the WHO Western Pacific and South-East Asia regions from 1999 to 2019 [3]. Singapore faces a particularly urgent challenge, where 1 in 3 Singaporeans aged 18–74 y were reported to have hypertension in 2020, and 9 in 10 Singaporeans exceeded the daily recommended sodium intake levels in 2022 [4,5].

Despite global recognition of the sodium crisis, addressing it remains difficult due to a range of multifaceted, persistent behavioral barriers. Commonly cited barriers include one’s accustomization to salty diets or taste preference for salt, deep-seated misconceptions about salt or sodium, and limited knowledge on recommended sodium intake and sources, which, when compounded by broader contextual factors, hinders both individuals and their networks from adopting and maintaining lower-sodium behaviors [6,7]. Similar concerns regarding sodium-related knowledge, attitudes, and behaviors (KAB) were also identified in Singapore, as evidenced by a 2020 formative study [8]. Although organizational and industry-level measures such as product reformulations [9] and education efforts are in place [10,11], these approaches have either relied largely on passive dissemination of informational resources catered to the mass majority or often resource-intensive clinic-based educational models. There is thus a crucial need to explore novel models of promoting sodium awareness and behavior change in a tailored, impactful, and sustainable way.

Recent evidence highlights the powerful potential that family members harness to facilitate impactful dietary behavior change [[12], [13], [14]]. Two common models of family-based interventions are family-wide and family-led interventions. Although the former, where all family members are direct intervention recipients, have shown promise within Asian communities [15], intervening and coordinating with multiple family members are often significantly more resource intensive and logistically challenging. Comparatively, family-led interventions see that only 1 family member directly receives the intervention, whereas other family members receive the intervention indirectly from this family member. Leveraging existing familial ties, family-led models have shown success across various health domains. For example, young Vietnamese American adults were trained as family health advocates to share cancer screening information through private group chats [16], and educational campaigns have empowered families to promote hepatitis B screening within Asian communities [17]. These approaches are especially powerful when targeting older adults, who often rely on family for health-related decisions [[18], [19], [20]]. Similar findings have also been documented in the dietary landscape, as evidenced by a child-led intervention, which showed promising potential in indirectly fostering reduction of salt intake across different generations of the family through a digital-based intervention, irrespective of their coresidence status [21]. These findings thus demonstrate that although young adults themselves often may not be directly in charge of cooking or food-related decisions in household settings, frequent interaction with those who make key dietary decisions position them as effective conduits for knowledge and behavior change [22]. Moreover, young adults are generally more receptive to diverse diets, particularly in their willingness to explore new foods and eating habits as compared with older adults [14,23]. Coupled with their unique positioning within multigenerational households, young adults (especially in Asian or immigrant communities) often face fewer logistical, linguistic, and technological barriers to participating in structured interventions, as they are more likely to have attained higher levels of formal education, which is associated with stronger digital literacy and language proficiency, particularly in contexts where interventions are delivered in English or via digital content [14,16,24]. These factors make young adults a promising and practical entry point for promoting sodium reduction across families, combining feasibility with high potential for impact [14].

Understanding key mechanisms by which young adults initiate, promote, and sustain dietary behavioral change is crucial such that these insights can be leveraged to develop adaptable approaches to behavior change. The Theory of Planned Behavior (TPB) and Family Systems Theory (FST) provide useful frameworks for understanding behavior change in family contexts. TPB highlights the roles of attitudes (beliefs about outcomes), perceived behavioral control (PBC; confidence in one’s ability), and subjective norms (perceived social expectations), and has been widely applied in dietary interventions [25], including among young adults [26]. FST supports the premise of family-oriented approach to health by viewing the family as an interdependent system where members influence one another through communication, roles, and hierarchies [27]. Pertaining to behavior change in family contexts specifically, a qualitative study identified 11 distinct mechanisms by which young adults influence family members, with their extent of use shaped by household structure and interaction frequency [22]. For instance, in close-knit households where communication is potentially more frequent and open, young adults may feel more comfortable in utilizing facilitating strategies such as knowledge sharing or encouragement to build behavioral capability and reinforce expectations around sodium reduction [13]. In more hierarchical families, where elders usually have more authority and young adults possess less influence or are more afraid to directly offer differing dietary suggestions or opinions (especially where older members have ingrained dietary habits), shaping strategies like modifying the food environment to foster self-efficacy and support healthier choices may be more effective [13]. These mechanisms help to explain variation in family influence and, when viewed through frameworks such as TPB and FST, provide a strong foundation for family-led dietary interventions.

Despite the widely acknowledged role of family members in shaping dietary behaviors, family-led dietary behavior change has not been systematically explored and evaluated in Singapore. This pilot intervention aimed to test the feasibility of implementing a cocreated, online, young adult-led sodium-reduction program [28], and to explore its practical potential to foster healthier sodium-related behaviors across the family in a multiethnic, socially diverse Asian context. This article specifically reports on the pilot’s primary outcomes by examining changes in sodium-related KAB among young adults and their family members.

## Methods

### Study design

A pre-post, single-group proof-of-concept study was conducted to pilot-test a co-created sodium-reduction educational intervention in Singapore [28], from August 2024 to January 2025. The study lasted a total of 6 wk for each participant, involving a 2-wk educational and a 4-wk implementation phase. The young adult-led intervention aimed to improve sodium-related KABs of both young adults and their family members, although fostering the development of strategies for sodium reduction across the family. By leveraging familial bonds, young adults acted as change agents, spearheading efforts to support and foster behavioral changes within their family unit, although family members were indirect intervention recipients.

### Participants

English-speaking Singaporean citizen or permanent resident (PR) young adults, defined as aged 21–35 y [29], along with family members whom they ate, cooked, or grocery shopped with weekly, were eligible to participate in this study. Recruitment occurred via outreach efforts on email listservs and various social media and messaging platforms popular among young adults (i.e., university associations, Instagram, Telegram). Interested young adults completed an online screener survey form to confirm eligibility. The research team then contacted interested individuals through purposive sampling to optimize representation across diverse genders and ethnicities (particularly among the major ethnic subgroups of Singapore: Chinese, Malay, and Indian families) and family structures (including participants who only had 1 or 2 family contacts and those with 3 or more). A brief consent call was administered, after which informed written consent was obtained and documented electronically from young adults.

Recruited young adults were then asked to invite minimally one, but ≤3 family members (i.e., parents, siblings, grandparents, aunts/uncles, cousins, spouses, parent-/sibling-in-laws) aged ≥21 y, English speaking, Singaporean/PR, who eat, cook, or grocery shop with the young adult at least weekly, to also participate. Individuals aged ≥65 y must not demonstrate cognitive impairments, as measured through a staff-administered abbreviated mental test [30]. Although family members were not recipients of the main intervention content, they were still considered research participants due to their involvement in outcome assessments and implementation activities. Thus, invited family members were first contacted and, if interested, asked to complete a similar screener survey form and consent call, following which informed written consent was obtained. Importantly, minimally one invited family member must participate, before a young adult was deemed enrolled in the study. All participants provided informed consent and were reimbursed accordingly for their time in 2 payments, distributed across different phases of the study. Ethics approval was obtained from the National University of Singapore Institutional Review Board (NUS IRB; NUS-IRB-2024-143).

### Intervention

**FIGURE 1:**
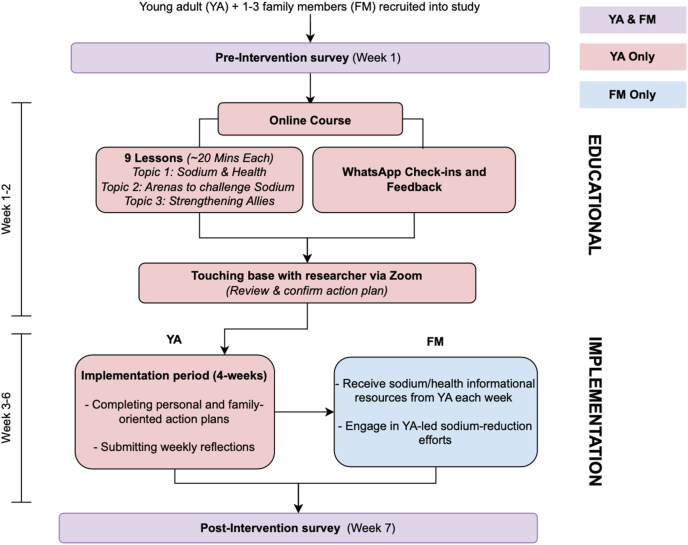
Flow diagram outlining the Supporting Household heAlth through familY-led Promotion (SHAYP) intervention for young adult (YA) and family member (FM) participants.

**FIGURE 2:**
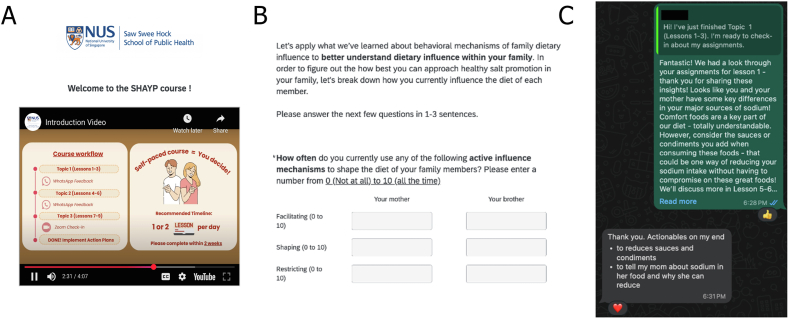
Snapshots of (A) video lesson, (B) assignment question, and (C) WhatsApp feedback.

All consented young adults and family members first completed a 30-min preintervention survey online via Qualtrics, either self-administered or completed with staff or family assistance. Young adult participants then entered the educational phase (Figure 1), featuring a self-paced sodium-reduction educational course delivered through Qualtrics. The course comprised 9 videos grouped into 3 topics: *1*) Sodium & Health (guided by attitude construct [TPB]), *2*) Arenas to Challenge Sodium (guided by PBC construct [TPB]), and *3*) Strengthening Allies (guided by subjective norms construct [TPB and FST]) and was cocreated with academic researchers and young adults (end-users, nonparticipants of this study), with development details outlined in a separate publication [28]. Briefly, these videos served to *1*) provide foundational knowledge and clarify misconceptions of sodium, although highlighting the consequences of excessive consumption; *2*) introduce common sources of sodium and key sodium-reduction strategies when eating out, grocery shopping, and cooking; *3*) introduce behavior change mechanisms that young adults can employ to promote household-wide sodium reduction. After each video, participants completed short-answer and free-response assignments (Figure 2) designed to reinforce key concepts and contextualize course content into their own lives. These assignments guided young adults in assessing personal and family sodium-related behaviors and in developing tailored action plans, supported by researcher feedback and Specific, Measurable, Attainable, Relevant, Time-based (SMART) goal setting [31]. Of note, the intervention was delivered in a structured but flexible and nonrestrictive manner such that young adults have agency to adapt and implement course content according to their unique family dynamics. To minimize participant burden, videos were brief (∼3–5 min each), and assignments took ∼10–15 min, with each topic requiring at most an hour to complete. Although participants were required to progress sequentially (ensured by password-protected topics) they had up to 2 wks to complete the course and were encouraged to follow a recommended pace of one lesson per day. With the intention for convenient access, accommodation of different availabilities, and fostering a sense of autonomy (all critical for intervention adherence and content retention [32,33]) participants had flexibility in setting their own schedule of lessons.

The research team provided tailored feedback based on participants’ assignment responses after the completion of Topics 1 (Lessons 1–3) and 2 (Lessons 4–6) via messages in WhatsApp (with message logs tracked and maintained to ensure that all participants received the intervention content as intended), ensuring continued engagement and guiding them in crafting their action plans. These plans required participants to develop both personal and family-oriented sodium-reduction goals. Participants identified whether their goals related to eating out, shopping for food, or cooking/preparing meals and, for family-oriented goals, specified whether they would directly participate in the behavioral focus (e.g., making a change when they eat out, cook/prepare meals, or shop for food with or for their family member) or if they would not directly participate (e.g., making a change when the family member eats out, cooks/prepares meals, or shops for food for themselves). Additionally, participants categorized their goals based on FST-guided behavioral change mechanisms (active influence), defined and introduced during the course as intentional efforts to shape the diet of a family member, which may include facilitating (encouraging sodium-reduction behaviors without enforcement or discouraging high-sodium dietary habits), shaping (taking initiative on another’s behalf, such as buying or preparing meals to make sodium reduction easier or high-sodium foods less accessible), and restricting (limiting or controlling high-sodium food consumption or enforcing sodium reduction through rewards or penalties) [22]. Notably, family members did not receive any direct intervention or participate in the course, although young adults were encouraged to consult with family members as part of developing the family-based needs assessment to inform the development of their action plans. Ultimately, participants retained full autonomy in determining both their own and their family members’ level of engagement.

After completing the educational course, participants scheduled a 30-min post-course Zoom call with the research team to review and refine their action plans through in-depth discussions based on their assignment responses. To ensure fidelity to the intervention protocol, these calls were guided by a standardized topic guide to ensure consistency in goal-setting support, and summaries were recorded in field notes immediately after each session. Along with the updated action plans, participants received instructions for the implementation phase, which began immediately afterward and lasted 4 wk (Figure 1). During this phase, young adult participants put their action plans into practice, with progress tracked through weekly reflections submitted via Qualtrics, where they reported on their successes, challenges, and key facilitators (to monitor implementation). These reflections were reviewed weekly by the research team to monitor adherence and flag any major deviations from the planned activities. Although participants were required to submit ≥1 reflection for the implementation phase to be considered complete, automated weekly reminders were sent throughout the 4 wk to encourage engagement. At the end of the 4 wk, both young adult participants and family members completed a 20-min post-intervention survey via Qualtrics to assess outcomes and overall experiences.

Participants who showed inactivity at any study phase received reminders at 3-, 5-, and 7-d intervals. If they responded, they were allowed to continue, and extensions were granted for unforeseen circumstances if communicated. However, if inactivity persisted after the third reminder, participants were given the option to withdraw from the study. The terms of discontinuation were clearly explained, and those who agreed to withdraw were classified as “lost to attrition,” with the stage of termination recorded.

### Measures

#### Implementation-related outcomes

A wide range of implementation outcomes were measured to reflect how participants engaged throughout the program. The number of days required to complete the course, time spent on each topic of the course, and statistics representative of engagement with assignments (e.g., mean number of characters of text provided per assignment question) were tracked. As part of the assignments, participants indicated the goal’s domain (e.g., eating out, cooking, shopping) and the young adult’s involvement, i.e., whether the behavior change was aimed at actions the family member took independently (e.g., eating out alone) or those involving the young adult directly (e.g., eating out together). The weekly reflection survey completed during implementation also asked participants to rate, on a scale of 1–10, the following: the effort they had put in that particular week on their personal goal and family-oriented goal and how much success they had in meeting these goals. Additionally, which behavioral change mechanism(s) they employed for family members (facilitating, shaping, and restricting) and obstacles they faced in implementing goals (e.g., no/limited time, opportunities to eat out or cook, family interactions, or family not interested or accepting) were also identified.

#### Sodium-related and other survey outcomes

A questionnaire was administered both pre-intervention and post-intervention; sodium-related survey items have been displayed in Supplemental File 1. Of these items, participant demographic, dietary, and health information were also collected at the preintervention stage. These include age, sex, race, household income, type of housing, relationship status, individualism, and collectivism measured through the Vertical and Horizontal Individualism and Collectivism scale [34], self-rated health status and effort put into one’s health (from 1 to 10), the presence of cardiovascular health conditions (high cholesterol, hypertension, heart disease, or diabetes), and weight status (determined with BMI calculated from self-reported weight and height, using Asian-specific cutoffs for overweightness and obesity [35]). Diet healthfulness was measured through a 4-item, 7-point food frequency questionnaire. This was adapted from another local-based study, focused on key food groups representing common healthy and unhealthy dietary choices in Singapore [36], notably vegetables, fruits, fast food, and snacks. Although this initial, short questionnaire was developed for children, other dietary research on common foods consumed in Singapore was used to ensure that the food groups aligned with those commonly consumed among adult Singaporeans as well [37].

Both the pre-intervention and post-intervention questionnaires contained sections relevant to sodium-relevant knowledge and behaviors. Items included were adapted from an earlier survey developed for a Singaporean population [8]. The sodium knowledge section included 9 multiple choice items focusing on declarative knowledge related to sodium consumption (e.g., maximum recommended sodium and salt intake, foods high in sodium content); responses were then scored and scaled to generate a knowledge score from 1 to 10 (lowest to highest knowledge). The sodium behaviors section asked participants to indicate how frequently (from all the time to not even 1 time) they participated in 10 different sodium consumption–related behaviors in the last week (e.g., adding salt, sauces, or condiments at the table or while cooking, consuming foods labeled as “low sodium,” asking for meals to be prepared with no or less salt when eating out) (Supplemental File 1); responses were similarly scored and scaled to generate a behavior score from 1 to 10 (lowest to highest sodium-reducing behaviors).

Both questionnaires also contained a section, developed using TPB, aimed at assessing the constructs of attitudes (e.g., following a low-sodium diet will significantly improve my health), subjective norms (e.g., most people who are important to me think I should follow a low-sodium diet), PBC (e.g., I am confident in my ability to cook or prepare tasty, low-sodium meals), and behavioral intentions (e.g., I plan to follow a low-sodium diet in the next month) related to sodium reduction (Supplemental File 1). Question wordings were informed by past TPB-informed dietary questionnaires [25] and tailored to the content of the course. Participants were asked to rank their agreement with 19 different items related to the 4 TPB constructs on a scale of 1–10. Responses were scored and scaled accordingly for each construct to generate a score from 1 to 10 (weakest to strongest).

### Analysis

Descriptive analyses were conducted to summarize the sociodemographic characteristics and health indicators of young adult and family member participants. Engagement and implementation data were also analyzed descriptively, including the number of days required to complete the course, total time spent on the course, and assignment engagement metrics (e.g., mean text length of responses). Additionally, goal-setting data were examined to categorize personal and family-oriented goals, focusing on the primary food behaviors targeted (eating out, cooking, and grocery shopping) and the extent to which young adults were directly involved in the behaviors specified in their family-oriented goals.

Bivariable analyses were conducted to assess pre-post changes in sodium-related knowledge, behaviors, and TPB constructs, with paired *t* tests used to compare preintervention and postintervention outcomes for both young adults and family members. Multivariable linear mixed models (accounting for individual-level and family-level random effects) were then used to examine postintervention changes in sodium-related outcomes, adjusting for age, sex, race, income, baseline weight status, self-rated health, self-rated effort toward health, and dietary healthfulness. Key sociodemographic confounders were identified based on bivariable analyses and prior literature on dietary and health behavior determinants [38]. To account for multiple comparisons across data sets and outcomes, Bonferroni-adjusted *P* values were computed by multiplying raw *P* values by the total number of tests (*n* = 18). These corrected *P* values are presented alongside unadjusted values. Lastly, exploratory interaction analyses were conducted to assess whether greater engagement with the program (measured by total time spent on the course) and greater effort (measured by self-reported effort in achieving personal and family-oriented goals) were associated with differential changes in sodium-related outcomes among both young adults and family members. All statistical analyses were conducted using R (version 4.3.0, R Foundation for Statistical Computing).

## Results

Recruitment began in September 2024, with 178 eligible young adults completing the screener. Due to high demand, recruitment closed in October 2024. Of these, 39 young adults were contacted between September 2024 and January 2025, initially on a first-come, first-served basis, and subsequently through purposive sampling to include underrepresented gender, ethnicity, and family structure groups. One was deemed ineligible during consent obtainment following more information provided, and of the 38 remaining, 36 (94.7%) were successfully recruited and enrolled; one declined due to time constraints, and another was unable to recruit a family member. A total of 81 family members consented to participate, although one withdrew shortly after due to time constraints, leaving 80 family members. During the study, 35 of the 36 young adults completed both the intervention and implementation phases, whereas one dropped out at the implementation phase due to time constraints (2.7% attrition), resulting in the withdrawal of the accompanying family member (leaving 79 remaining). All remaining young adults and family members successfully completed the preintervention and postintervention surveys, yielding a final analytic sample of 35 young adults and 79 family members.

Young adult participants had a mean age of 24.4 y (SD: 3.1), were 57.1% female, and predominantly Chinese (74.3%), with most living in middle- to high-income households and 5-room or executive Housing & Development Board flats (Table 1). A large majority (80.0%) were not partnered or married. They reported relatively high individualism (mean: 28.7; SD: 3.8) and comparatively slightly lower collectivism (mean: 25.7; SD: 5.4), along with good self-rated health (mean: 7.29; SD: 1.34) and moderate effort put into maintaining health (mean: 6.51; SD: 1.48); only 2.9% reported a cardiovascular NCD. Family members had a mean age of 42.8 y (SD: 15.5), were similarly 57.0% female and 77.2% Chinese, with 65.8% partnered or married. They reported similar health (mean: 7.1; SD: 1.6) and effort scores (mean: 6.6; SD: 2.0) as young adults but a higher prevalence of cardiovascular NCDs (22.8%). Overweight or obesity was observed in 38.2% of young adults and 48.1% of family members. All young adults were living with the family members they participated in the study with, except for 9 cases: in 1 case, the young adult or participating sibling was temporarily staying in a school dormitory on weekdays, and in 8 cases, the family member did not live with them permanently (e.g., a participating sibling who had moved out after marriage or a participating extended relative such as an uncle, aunt, or cousin living in a different house).

**TABLE 1:** Characteristics of Singaporean young adults and family member participants (*n* = 114).

Variable	Total	Young adult *(n* = 35)	Family member (*n* = 79)	*P* value
Age, mean (SD)	37.2 (15.5)	24.4 (3.1)	42.8 (15.5)	<0.001
Sex, *n* (%)				
Female	65 (57.0)	20 (57.1)	45 (57.0)	0.999
Male	49 (43.0)	15 (42.9)	34 (43.0)	
Race, *n* (%)				
Chinese	87 (76.3)	26 (74.3)	61 (77.2)	0.800
Indian	17 (14.9)	5 (14.3)	12 (15.2)	
Malay/Other	10 (8.8)	4 (11.4)	6 (7.6)	
Household income, *n* (%)				
$2000–$5999	23 (20.9)	8 (23.5)	15 (19.7)	0.762
$6000–$9999	46 (41.8)	15 (44.1)	31 (40.8)	
≥$10,000	41 (37.3)	11 (32.4)	30 (39.5)	
Type of housing, *n* (%)				
1–3 room HDB	16 (14.0)	5 (14.3)	11 (13.9)	0.999
4-room HDB	26 (22.8)	8 (22.9)	18 (22.8)	
5-room / Exec HDB^1^	52 (45.6)	16 (45.7)	36 (45.6)	
Condominium / landed	20 (17.5)	6 (17.1)	14 (17.7)	
Relationship status, *n* (%)				
Not partnered or married	54 (48.6)	28 (80.0)	26 (34.2)	<0.001
Partnered or married	57 (51.4)	7 (20.0)	50 (65.8)	
Individualism (4–36), mean (SD)	27.5 (5.01)	28.7 (3.8)	26.9 (5.4)	0.086
Collectivism (4–36), mean (SD)	27.2 (6.3)	25.7 (5.4)	27.8 (6.6)	0.109
Health status, mean (SD)	7.1 (1.52)	7.3 (1.3)	7.1 (1.6)	0.450
Effort put in health, mean (SD)	6.6 (1.82)	6.5 (1.5)	6.6 (2.0)	0.776
Diet healthfulness (1–7), mean (SD)	4.6 (0.6)	4.4 (0.5)	4.7 (0.7)	0.064
Cardiovascular noncommunicable disease^2^				
No	95 (83.3)	34 (97.1)	61 (77.2)	0.018
Yes	19 (16.7)	1 (2.9)	18 (22.8)	
Weight status				
Underweight	13 (11.5)	5 (14.7)	8 (10.1)	0.848
Normal	49 (43.4)	16 (47.1)	33 (41.8)	
Overweight	37 (32.7)	10 (29.4)	27 (34.2)	
Obese	15 (13.2)	4 (11.4)	11 (13.9)	

Abbreviations: HDB, Housing & Development Board; SD, standard deviation.

1 Executive HDB refers to public housing with similar layouts and number of rooms to a 5-room HDB but with a larger living room area or space.

2 High cholesterol, high blood pressure, heart disease, or diabetes.

**FIGURE 3:**
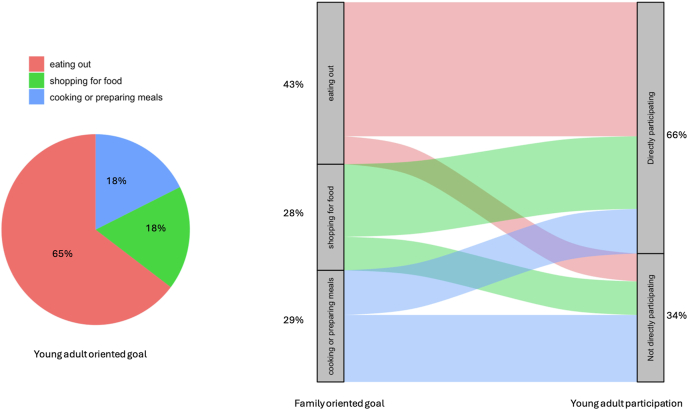
Focus of goals young adults made to reduce their own and their family member’s sodium intake.

Young adults took a mean of 7.74 d (SD: 6.85) to complete the course. Regarding goal setting (Figure 3), 65% of personal goals crafted by young adults focused on eating out, whereas the remaining 35% were evenly split between goals related to cooking/preparing meals and grocery/food purchases. Similarly, family-oriented goals also leaned toward eating out (43%), followed by cooking/meal preparation (29%) and grocery shopping (28%). As shown in Figure 3, young adults who developed family-oriented goals related to eating out often played direct roles in the targeted behavior. An example of such a goal was “*When I eat out with my parents, my goal is to order ≥1* *lower-in-sodium* *option on their behalf such as foods with healthier cooking methods or with less gravy*.” In contrast, for family-oriented goals related to cooking or food shopping, participants were more likely to play an indirect role in shaping behaviors. An example of such a goal was: “*When my mother cooks or prepare[s] meals, my goal is to encourage her to use* *lower-sodium* *seasonings or natural spices*.”

Weekly reflections indicated that young adults invested more effort into achieving their personal goals (mean: 6.5, SD: 1.8) compared with their family-oriented goals (mean: 5.5, SD: 2.1). The degree of success in meeting both goal types was proportional to the effort invested and remained consistent throughout the month (Table 2). Among the 3 influence mechanisms used for family-oriented goals, *facilitating* was the most commonly employed, with all participants utilizing it, followed by *shaping* (85.7%), whereas *restricting* was the least used (42.9%). Among potential barriers to goal implementation, participants indicated that limited opportunities to cook and a lack of time were the 2 most significant challenges.

**TABLE 2:** Reflections provided by young adult participants during weekly implementation of their personal and family goals.

	Overall	Week 1	Week 2	Week 3	Week 4
Effort put into meeting goal					
Personal goal, mean (SD)	6.6 (1.8)	6.4 (2.2)	6.2 (2.3)	6.8 (2.3)	6.8 (2.4)
Family goal, mean (SD)	5.5 (2.1)	5.2 (2.8)	5.3 (2.5)	5.8 (2.3)	5.8 (2.6)
Success in meeting goal					
Personal goal, mean (SD)	6.6 (1.8)	6.0 (2.4)	6.7 (2.4)	6.9 (2.3)	6.8 (2.5)
Family goal, mean (SD)	5.2 (2.0)	4.5 (2.6)	5 (2.6)	5.7 (2.3)	5.9 (2.6)
Family influence mechanisms used					
Facilitating^1^, yes (%)	34 (100)	26 (76.5)	23 (67.7)	29 (85.3)	28 (84.9)
Shaping, yes (%)	29 (85.3)	13 (38.2)	23 (67.7)	21 (61.8)	20 (60.6)
Restricting, yes (%)	15 (44.1)	7 (20.6)	7 (20.6)	8 (23.5)	5 (15.2)
Obstacles in implementing goal					
No/limited time, mean (SD)	4.6 (2.3)	5 (3.2)	4.5 (3.2)	4.9 (3.1)	3.9 (2.7)
No/limited opportunities to eat out, mean (SD)	3.4 (1.9)	3.7 (2.8)	3.2 (2.6)	3.4 (2.8)	3.2 (2.6)
No/limited opportunities to cook, mean (SD)	4.8 (2.5)	5.8 (3.3)	4.8 (3.3)	4.7 (3.6)	3.9 (3.1)
No/limited family interactions, mean (SD)	4.5 (2.3)	4.9 (3.3)	4.4 (3.1)	5.1 (3.3)	3.6 (2.8)
Family not interested / accepting, mean (SD)	2.4 (1.8)	2.8 (2.6)	2.3 (2.1)	2.5 (2.3)	2.2 (1.8)

Abbreviation: SD, standard deviation. Range: 1–10.

1 Facilitating (i.e., encouraging certain dietary behaviors without enforcement, or shaming dietary behaviors perceived as nondesirable), shaping (i.e., making foods specifically to align or reinforce the young adult’s preferences, or sharing food or cooking-related resources to support certain eating behaviors), restricting (i.e., restricting or controlling the consumption of specific foods, or enforcing dietary behaviors through rewards or punishments).

The bivariable analyses (Table 3) showed significant post-intervention improvements in sodium-related knowledge, behaviors, PBC, subjective norms, and behavioral intentions for both young adults and family members. In Bonferroni-corrected analyses, improvements in attitudes were not observed for either young adults or family members.

**TABLE 3:** Descriptive pre- and postintervention changes in sodium outcomes following intervention (*n* = 116).

	Preintervention	Postintervention	*P* value	Adjusted^1^*P* value
Sodium-related knowledge score				
Overall	4.95 (1.00)	6.06 (1.38)	<0.001	<0.001
Young adults	5.28 (0.82)	6.89 (1.11)	<0.001	<0.001
Family members	4.81 (1.04)	5.69 (1.34)	<0.001	<0.001
Sodium-related behaviors score^2^				
Overall	5.06 (1.50)	6.42 (1.33)	<0.001	<0.001
Young adults	4.77 (1.38)	6.59 (1.28)	<0.001	<0.001
Family members	5.19 (1.54)	6.35 (1.35)	<0.001	<0.001
TPB – attitudes score				
Overall	6.06 (1.5)	6.62 (1.35)	0.003	0.050
Young adults	5.76 (1.32)	6.67 (1.29)	0.017	0.306
Family members	6.19 (1.56)	6.59 (1.39)	0.054	0.980
TPB – PBC score				
Overall	5.61 (1.83)	7.67 (1.28)	<0.001	<0.001
Young adults	5.35 (1.79)	7.90 (1.20)	<0.001	<0.001
Family members	5.72 (1.85)	7.56 (1.31)	<0.001	<0.001
TPB – subjective norms score				
Overall	5.62 (1.98)	7.03 (1.56)	<0.001	<0.001
Young adults	5.27 (1.86)	6.95 (1.25)	<0.001	0.004
Family members	5.77 (2.03)	7.07 (1.69)	<0.001	<0.001
TPB – behavioral intentions score				
Overall	6.53 (2.36)	7.81 (1.64)	<0.001	<0.001
Young adults	6.63 (2.39)	8.11 (1.49)	0.002	0.034
Family members	6.49 (2.36)	7.68 (1.70)	<0.001	<0.001

Abbreviations: PBC, perceived behavioral control; TPB, Theory of Planned Behavior. Range: 1–10.

1 Bonferroni corrected to adjust for multiple comparisons.

2 Score reflects 10 sodium-specific behaviors (e.g., using less salt when cooking, avoiding adding salt at the table, choosing low-sodium products, checking food labels for sodium), details of which are described in Supplemental File 1.

**FIGURE 4:**
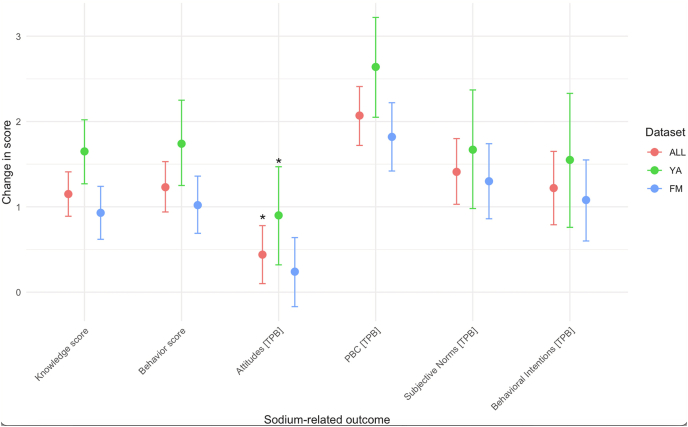
Adjusted^1^ postintervention changes in sodium-related knowledge, attitudes, and behaviors (*n* = 114). ^1^Adjusted for age, sex, race, income, baseline obesity status, self-rated health, self-rated effort put into health, and dietary healthfulness. Exact adjusted coefficients and 95% confidence intervals for each outcome and subgroup are reported in Supplemental Table 1. ∗No longer significant after Bonferroni correction to adjust for multiple comparisons. FM, family member; PBC, perceived behavioral control; TPB, Theory of Planned Behavior; YA, young adult.

In the multivariable adjusted analyses (Supplemental Table 1, Figure 4), PBC showed the greatest post-intervention improvement (+2.07, 95% confidence interval [95% CI]: 1.72, 2.41; *P* < 0.001), with not only a stronger effect among young adults (+2.64, 95% CI: 2.05, 3.22; *P* < 0.001) but also significant improvements for family members (+1.82, 95% CI: 1.42, 2.22; *P* < 0.001). Sodium-related knowledge (+1.15, 95% CI: 0.89, 1.41; *P* < 0.001) and behaviors (+1.23, 95% CI: 0.94, 1.53; *P* < 0.001) also improved in both groups, although young adults exhibited the largest gains (+1.65 and +1.74, respectively). Although attitudes were observed to improve post-intervention, mainly driven by improvements among young adults, they were not statistically significant.

Interaction analyses revealed that young adults who spent more time on the course (7 or more hours) experienced greater post-intervention improvements in attitudes toward sodium reduction (β-interaction = +1.51, *P* = 0.038). However, self-reported effort in achieving personal goals was not associated with a differing level of impact on any outcomes. Similarly, for family members, neither the time young adults spent on the course nor the effort reported in achieving family-oriented goals was linked to different levels of post-intervention change in any outcomes.

## Discussion

This study evaluated the preliminary impact of an online, young adult-led sodium-reduction intervention and offers valuable insights into the feasibility of using self-paced digital platforms for dietary behavior change. By examining engagement metrics alongside pre-post changes in sodium-related KABs within Singapore’s multicultural context, the findings demonstrate the potential of young adults to effectively drive household-level change using digital tools. The results also shed light on how young adults engage with and apply specific behavioral mechanisms to initiate and promote sodium reduction within families. These insights have important implications for the future design of dietary interventions, emphasizing the need for culturally responsive, flexible, and low-burden approaches that align with individual preferences and family dynamics. Taken together, these preliminary findings lay important groundwork for larger, scaled-up studies to more rigorously assess impact and long-term effectiveness.

Both personal and family-oriented goals were primarily centered on eating out. Although this is expected for personal goals, given young adults’ well-documented preferences for convenience and fast food [39,40], the prominence of eating out in family-oriented goals is more surprising. This contrasts with the existing literature, which typically locates family-based nutritional interventions within home settings, such as shared cooking or dining at home [41,42]. In Singapore, 40% of locals eat out several times a week [43] and only 22% cook at home daily [44], likely reflecting cultural preferences of food courts (i.e., hawker centers) offering convenient, affordable options for diverse groups, including for family meals, where 67% of Singaporeans dined out with their families [39,45]. Moreover, young adults were more personally and directly involved in family-oriented eating-out goals (e.g., participating in healthier behaviors with them) than in cooking or grocery purchasing–related goals, where behavioral health promotion was often more of light touch and encouragement oriented (i.e., “facilitating”). This may stem from lower self-efficacy in cooking and shopping, due to limited involvement in traditional family food roles [22]. Asian cultural values and hierarchies emphasizing elder respect [46] may also make such light-touch approaches generally more acceptable, as more direct and effort-intensive strategies (i.e., “shaping” or “restricting”) require greater familiarity with the recipient’s habits and can feel overly directive. Liu et al. [47] further noted that strategies used by elders to influence youth differ from those used by youth to influence elders, in part driven by these social norms. These insights thus highlight the importance of tailoring interventions to local culture and environment, and especially of aligning family-led approaches with the abilities, preferences, and dynamics of the designated change agents.

Unlike other education-based sodium-reduction interventions reporting notable improvements in sodium-related attitudes [48,49], our intervention did not associate with significant change, likely because participants were already aware and possessed some knowledge of the health risks associated with high-sodium intake, as supported by past research [8,50,51] as well as an ongoing qualitative study involving interviews with participating young adults and family members [52]. This was anticipated and thus, leveraged to directly inform our intervention design, which deliberately focused less on changing attitudes and more on offering practical, low-effort, and minimally disruptive strategies to reduce sodium. In contexts like Singapore, where awareness is already high, light-touch interventions may be more effective when they prioritize ease, feasibility, and integration into daily routines. Although such approaches may be less likely to shift attitudes, which are often resistant to change and may require more intensive strategies, they can still meaningfully support behavior change by addressing motivational and structural barriers (e.g., highlighting that lower-sodium salt can be easily found and purchased in frequently visited grocery stores, sending recipe suggestions via messaging platforms or social media that feature flavorful but low-sodium dishes using accessible ingredients). These findings underscore the value of tailoring interventions not only to the population’s baseline awareness but also to the appropriate level of intensity: prioritizing practical, habit-oriented strategies in more aware populations, and reserving more intensive, persuasive efforts for those with lower awareness or more resistant attitudes.

Among all TPB constructs measured, PBC showed the greatest improvement, with knowledge, intentions, behaviors, and subjective norms also demonstrating moderate gains. Although positive changes in PBC are not uncommon following sodium-reduction interventions [48,53], the extent of change observed in our study was notable. This may be partly due to our intervention’s efficacy in addressing common knowledge gaps prominent in Singapore, particularly around procedural sodium-reduction strategies [8]. Moreover, our intervention’s unique provision of an array of strategies relevant to the multiple food-relevant behaviors or settings, instead of solely focusing on a single aspect (e.g., cooking at home) as sometimes explored in other nutritional education interventions [41], could also explain the improvement in overall PBC observed. This flexibility, combined with an implementation period, may have enabled participants to apply what they learned, experience small successes, and build confidence—a positive feedback loop that strengthened PBC. Given the known challenges in sustaining dietary behavior change postintervention [49], and growing evidence of PBC’s central role in mediating intentions and behaviors [54,55], these findings highlight the need for future interventions to prioritize building PBC and to include structured opportunities for practice and reflection to support long-term adherence.

Although young adults reported investing more effort in their personal goals than in family-oriented ones, there was no significant association between sodium-related outcomes and course engagement metrics or goal effort. Despite these aligning with a systematic review showing inconsistent links between engagement and intervention efficacy [56], some studies have demonstrated that engagement with digital health interventions (along with characteristics reflective of degree of effort required or invested into the program) is associated with greater improvements in intervention outcomes [[57], [58], [59]]. Our findings suggest that digital interventions designed to be of low-effort, flexible, and self-paced may still be able to provide improvements in outcome, particularly for young adults, who prefer accessible, time-efficient intervention formats [60] and generally hold positive views toward web-based health programs [61]. Moreover, postintervention interviews, currently being explored qualitatively in a separate analysis, indicated that many family members adopted sodium-reduction behaviors to support the young adult’s role as a change maker, suggesting that household change was often not exclusively driven by the young adult’s efforts alone but became a shared family effort. Nonetheless, further research is needed to clarify how engagement influences outcomes in longer interventions and across a wider range of health behaviors, as well as to identify more objective ways of assessing effort (such as through regular action plan logs or behavioral tracking). Still, these preliminary findings suggest that engagement and effort may influence outcomes in varied ways, with individuals differing in how they absorb and implement content, especially when supported by flexible intervention formats and social reinforcement within the household.

This study has several strengths, including the novel application of a young adult-led, family sodium-reduction intervention within a multicultural Asian context, the use of a co-created, flexible digital platform aligned with young adult preferences, and high completion rates with minimal attrition. Grounding the intervention in behavioral and family systems theories further enhance its relevance and potential for scalability. However, several limitations must be acknowledged. Potential selection bias was present as the participant sample was largely composed of educated, health-conscious individuals recruited through school and social media channels, limiting generalizability to more diverse or less health-engaged populations. Although many households include family members with limited English skills, young adults in Singapore are typically bilingual and fluent in English [62], and the intervention leverages this bilingualism by enabling them to share English materials in ways that suit their families. Unlike most Asian immigrant contexts, there is no sizable non–English-speaking young adult population in Singapore, so language-related limitations are minimal. However, findings may differ for less health-conscious individuals (who may have competing priorities or lower perceived urgency) and for lower-income households (who may face cost or access barriers to adopting and sustaining lower-sodium choices). The absence of a control group and reliance on self-reported KABs limit causal inference, suggesting that the observed changes should be interpreted with caution as indicative trends rather than firm causal conclusions. Additionally, engagement and efforts were assessed primarily through self-report and digital metrics, which may not fully capture implementation behaviors, and similarly may be impacted by potential recall and/or social desirability bias. Moreover, the lack of physiological measures (e.g., blood pressure, urinary sodium, anthropometric measurements) restricts conclusions about clinical impact. Future studies should thus expand recruitment to more diverse populations, incorporate objective outcome and effort measures, and be scaled up as a randomized controlled trial with physiological end points to more rigorously assess impact and long-term effectiveness. Lastly, although young adults in our study were positioned as change agents within their families, many focused primarily on their individual goals rather than broader household change. This underscores the importance of complementing individual-level strategies with approaches that address other levels of socioecological influences on family dietary behavior (e.g., other sociocultural and interpersonal drivers, such as religiously driven eating patterns, influences from friends, as well as environmental and policy-level factors, such as food pricing, availability of lower-sodium options). Future studies may thus benefit from building upon our findings and integrating these layers to more effectively support family-wide change.

In conclusion, this pilot study demonstrated the feasibility and preliminary impact of adopting an online, young adult-led educational approach to improve sodium-relevant KABs at both individual and household levels. Actionable insights for developing culturally relevant, resource-efficient, low-effort, yet effective interventions were emphasized in this study, but future efforts to further explore any facilitators and/or barriers faced by both young adults and their family members in utilizing the mechanisms introduced and subsequently, in translating behavioral intentions into sustained sodium-reduction behaviors are beneficial. Addressing these aspects will help future studies to enhance the impact of family-led digital health interventions.

## Author contributions

The authors’ responsibilities were as follows: KMYL: methodology, formal analysis, investigation, data curation, writing - original draft; CMJC: methodology, writing - review & editing; IYHA: methodology, writing - review & editing; MFFC: methodology, writing - review & editing; SHA: conceptualization, methodology, formal analysis, writing - original draft, funding acquisition, supervision; and all authors have read and approved the manuscript.

## Funding

This study was funded by the National University of Singapore Saw Swee Hock School of Public Health Seed Fund for Ground-Up Research.

## Conflict of interest

The authors report no conflicts of interest.
