# Supplementary material for: Empowering Young Adults as Agents of Household Dietary Change: Findings From a Pilot Study Involving a Digital, Family-Led Sodium Reduction Intervention in Singapore
Source: Curr Dev Nutr. 2025 Aug 5;9(9):107523. doi: 10.1016/j.cdnut.2025.107523 (PMC12423401; doi:10.1016/j.cdnut.2025.107523)
Supplement: Multimedia component 1 [file mmc1.docx]

**Supplemental File 1: Subset of Pre- and Post-intervention Survey Questions Relevant to Sodium Knowledge, Attitudes, Behaviors**

Declarative knowledge on sodium (5 items)

1. When mentioned about "sodium", sometimes we hear about the term "salt" too. Which of these statements best describes the relationship between salt and sodium? *[Scores in brackets ()]*

- They are completely unrelated *(0)*
- They are exactly the same *(0)*
- Salt contains sodium *(1)*
- Sodium contains salt *(0)*
- Don't know *(0)*

1. Here is a list of food items, please select which food items you think are high in sodium content. (Select all that apply) *[Scores in brackets ()]*

- Whitebread *(0)*
- Cake/ brownies/ muffins *(0)*
- Candy *(0)*
- Canned soup *(1)*
- Fast-foods (e.g., hamburger, pizza) *(1)*
- Grains/ pasta/ rice *(0)*
- Instant noodles with seasoning powder *(1)*
- Luncheon meat/ hot dogs/ sausages *(1)*
- Fish ball, fish cake, crab stick *(1)*
- Corn flakes *(1)*
- Savoury snacks (e.g., chips, crackers) *(1)*
- Sliced cheese *(1)*
- Oyster sauce *(1)*
- Soy sauce *(1)*
- Chilli sauce *(1)*
- Ketchup *(1)*

1. What do you think is the maximum recommended amount of salt an average, healthy individual should consume in one day? *[Scores in brackets ()]*

- 10g (2 teaspoon) *(0)*
- 5g (1 teaspoon) *(1)*
- 2g (1/2 teaspoon) *(0)*
- Don't know *(0)*

1. What do you think is the maximum recommended amount of sodium an average, healthy individual should consume in one day? *[Scores in brackets ()]*

- 3000 mg *(0)*
- 2500 mg *(0)*
- 2000 mg *(1)*
- 1500 mg *(0)*
- Don't know *(0)*

1. To what extent do you agree or disagree with the following statements? Please make sure you select an option for each line. *[Scores in brackets (), *Missing responses scored (0)]*

|  | Strongly disagree | Somewhat disagree | Neither agree nor disagree | Somewhat agree | Strongly agree | Don’t know |
| --- | --- | --- | --- | --- | --- | --- |
| Sodium is only one component of table salt, and comprises just 40% of its weight. | □  *(0)* | □  *(0)* | □  *(0)* | □  *(1)* | □  *(2)* | □  *(0)* |
| Himalayan salt, pink salt, sea salt, and gourmet salts are healthier than regular table salt. | □  *(2)* | □  *(1)* | □  *(0)* | □  *(0)* | □  *(0)* | □  *(0)* |
| MSG is an example of a recommended low-sodium alternative for salt. | □  *(0)* | □  *(0)* | □  *(0)* | □  *(1)* | □  *(2)* | □  *(0)* |
| Frozen vegetables are healthier than canned vegetables. | □  *(0)* | □  *(0)* | □  *(0)* | □  *(1)* | □  *(2)* | □  *(0)* |
| You can train your taste buds to be more sensitive to salt, letting you enjoy salty flavours without needing too much salt. | □  *(0)* | □  *(0)* | □  *(0)* | □  *(1)* | □  *(2)* | □  *(0)* |

Recent behaviors related to sodium consumption (10 items)

In the last week, how often, if at all, did you do the following? Please make sure you select an option for each line. *[Scores in brackets (), *Missing responses scored (0)]*

|  | All of the time | Most of the time | Half of the time | Some of the time | None of the time | Not applicable / Did not occur |
| --- | --- | --- | --- | --- | --- | --- |
| Add salt, sauce, or condiments (e.g. soy sauce, liquid seasoning, oyster sauce, chilli sauce) **at the table**. | □  *(-2)* | □  *(-1)* | □  *(0)* | □  *(1)* | □  *(2)* | □  *(0)* |
| Add salt, sauce, or condiments (e.g. soy sauce, liquid seasoning, oyster sauce, chilli sauce) **when cooking**. | □  *(-2)* | □  *(-1)* | □  *(0)* | □  *(1)* | □  *(2)* | □  *(0)* |
| Use spices/herbs instead of salt and condiments when cooking. | □  *(2)* | □  *(1)* | □  *(0)* | □  *(-1)* | □  *(-2)* | □  *(0)* |
| Made an effort to buy/eat less snacks. | □  *(2)* | □  *(1)* | □  *(0)* | □  *(-1)* | □  *(-2)* | □  *(0)* |
| Made an effort to eat less pickled or preserved foods. | □  *(2)* | □  *(1)* | □  *(0)* | □  *(-1)* | □  *(-2)* | □  *(0)* |
| Made an effort to eat less pre-made/ ready-to-eat/ processed foods. | □  *(2)* | □  *(1)* | □  *(0)* | □  *(-1)* | □  *(-2)* | □  *(0)* |
| Made an effort to eat less fast-food (e.g., McDonalds, KFC, Pizza Hut). | □  *(2)* | □  *(1)* | □  *(0)* | □  *(-1)* | □  *(-2)* | □  *(0)* |
| When eating out, asked to have your meal prepared with no or less salt and condiments. | □  *(2)* | □  *(1)* | □  *(0)* | □  *(-1)* | □  *(-2)* | □  *(0)* |
| Looked at a food label to check the sodium content of a food item. | □  *(2)* | □  *(1)* | □  *(0)* | □  *(-1)* | □  *(-2)* | □  *(0)* |
| Consumed foods labelled "sodium free", "low sodium" or "reduced sodium". | □  *(2)* | □  *(1)* | □  *(0)* | □  *(-1)* | □  *(-2)* | □  *(0)* |

Attitudes towards sodium and salt and Theory of Planned Behavior questions (17 items)

How much do you agree with the below statements on a scale of 1 (Strongly Disagree) to 10 (Strongly Agree)? *[Statements were scored positively according to rank level (i.e. 1 to 10; 1 = 1 point), *Statements were negatively scored according to rank level (i.e. -1 to -10; 1 = -1 point)]*

- Attitudes
  - Following a low-sodium diet will significantly improve my health.
  - *Following a low-sodium diet is not as important as other priorities in my life.
  - *I believe salt is needed to make food tasty.
- Perceived behavioral control
  - I am aware of the recommended amount of sodium I should be consuming.
  - I am aware of the important sources of sodium in my life.
  - I am aware of strategies that I can use while grocery shopping to minimize my sodium intake.
  - I am aware of strategies that I can use while cooking or preparing meals to minimize my sodium intake.
  - I am aware of strategies that I can use while eating out to minimize my sodium intake.
  - I am confident in my ability to cook or prepare tasty, low-sodium meals.
  - I am confident in my ability to choose low-sodium foods at the grocery store.
  - I am confident in my ability to minimize my sodium intake when eating out.
  - I have the capacity to control the amount of sodium in my diet.
- Subjective Norms
  - It is expected of me that I follow a low-sodium diet in the next month.
  - Most people who are important to me think I should follow a low-sodium diet.
  - When it comes to following a low-sodium diet, I want to do what most people who are important to me want me to do.
  - The people in my life whose opinions I value follow a low-sodium diet.
- Behavioral Intentions
  - I will try to follow a low-sodium diet in the next month.
  - I plan to follow a low-sodium diet in the next month.
  - I intend to follow a low-sodium diet in the next month.

|  | **Estimate** | **95%CI** | **p value** | **Adjusted p value^2^** | **ICC** |
| --- | --- | --- | --- | --- | --- |
| **Sodium-related Knowledge score** |  |  |  |  |  |
| Overall | +1.15 | (0.89, 1.41) | <0.001 | <0.001 | 0.309 |
| Young adults | +1.65 | (1.27, 2.02) | <0.001 | <0.001 | 0.185 |
| Family members | +0.93 | (0.62, 1.24) | <0.001 | <0.001 | 0.289 |
| **Sodium-related Behaviors score ^3^** |  |  |  |  |  |
| Overall | +1.23 | (0.94, 1.53) | <0.001 | <0.001 | 0.274 |
| Young adults | +1.74 | (1.25, 2.25) | <0.001 | <0.001 | 0.096 |
| Family members | +1.02 | (0.69, 1.36) | <0.001 | <0.001 | 0.180 |
| **TPB - attitudes score** |  |  |  |  |  |
| Overall | +0.44 | (0.10, 0.78) | 0.013 | 0.238 | 0.163 |
| Young adults | +0.90 | (0.32, 1.47) | 0.008 | 0.144 | 0.167 |
| Family members | +0.24 | (-0.17, 0.64) | 0.250 | 0.999 | 0.206 |
| **TPB - PBC score** |  |  |  |  |  |
| Overall | +2.07 | (1.72, 2.41) | <0.001 | <0.001 | 0.248 |
| Young adults | +2.64 | (2.05, 3.22) | <0.001 | <0.001 | 0.096 |
| Family members | +1.82 | (1.42, 2.22) | <0.001 | <0.001 | 0.303 |
| **TPB - subjective norms score** |  |  |  |  |  |
| Overall | +1.41 | (1.03, 1.80) | <0.001 | <0.001 | 0.285 |
| Young adults | +1.67 | (0.98, 2.37) | <0.001 | 0.003 | 0.107 |
| Family members | +1.30 | (0.86, 1.74) | <0.001 | <0.001 | 0.340 |
| **TPB - behavioral intentions score** |  |  |  |  |  |
| Overall | +1.22 | (0.79, 1.65) | <0.001 | <0.001 | 0.345 |
| Young adults | +1.55 | (0.76, 2.33) | 0.002 | 0.035 | 0.055 |
| Family members | +1.08 | (0.60, 1.55) | <0.001 | <0.001 | 0.364 |

**Supplemental Table 1: Adjusted^1^ post-intervention changes in sodium-related knowledge, attitudes, and behaviors (n=116)**

^1^ Adjusted for age, sex, race, income, baseline obesity status, self-rated health, self-rated effort put into health, and dietary healthfulness. ^2^ Bonferroni corrected to adjust for multiple comparisons ^3^ Score reflects 10 sodium-specific behaviors (e.g., using less salt when cooking, avoiding adding salt at the table, choosing low-sodium products, checking food labels for sodium), details of which are described in Supplemental File 1.
